# A Dictionary of Practical Medicine; Comprising General Pathology, the Nature and Treatment of Diseases, Morbid Structures, &c.

**Published:** 1845-03

**Authors:** 


*A Dictionary of Practical Medicine; comprising General Pathology, the Nature and Treatment of Diseases, Morbid Structures, &c.* By James Copland, M. D., F. R. S. Edited, with additions, by Charles A. Lee, M. D. Part III. New York: Henry G. Langley.

We have the satisfaction of acknowledging the receipt of the three first *parts* of this valuable work. We have on former occasions indicated our high estimate of its merits, and we trust the profession will be supplied with the succeeding numbers, at least so far as the work has progressed in England, with the same regularity and despatch that have marked the progress of its kindred publication in this city.

